# Out-of-hospital cardiac arrests in Swedish nursing homes: occurrence, treatment, and survival compared to private residences

**DOI:** 10.1186/s13049-025-01496-y

**Published:** 2025-10-21

**Authors:** Åsa Mobaeck, Anders Bremer, Håkan Johansson, Jörg Carlsson, Johan Israelsson

**Affiliations:** 1 Department of Health and Caring Sciences, Faculty of Health and Life Sciences, Linnaeus University, Kalmar/Växjö, Sweden; 2 Department of Primary Care, Region Kalmar County, Kalmar, Sweden; 3 Department of Research, Region Kalmar County, Kalmar, Sweden; 4 Department of Internal Medicine, Kalmar County Hospital, Region Kalmar County, Kalmar, Sweden

**Keywords:** Cardiopulmonary resuscitation, Heart arrest, Nursing homes, Survival

## Abstract

**Background:**

In Sweden, most out-of-hospital cardiac arrests (OHCAs) occur in private residences and nursing homes. Although studies suggest that nursing home staff appear hesitant to start cardiopulmonary resuscitation (CPR) before ambulance staff arrive, it is unknown whether treatment and outcomes among those who suffer OHCA in nursing homes differ from private residences. The aim of the study was to describe OHCA occurrence, treatment, and 30-day survival in people aged 65 years or older in Swedish nursing homes, in comparison with private residences.

**Methods:**

This retrospective registry study utilized data from the Swedish Register of Cardiopulmonary Resuscitation from 1992 to 2022. The study included 59 459 OHCAs. Data were analyzed using descriptive and inferential statistics, complemented with generalized linear models.

**Results:**

The number of OHCAs was 56 379 in private residences and 3 080 in nursing homes. While the occurrence of OHCA increased in private residences it remained stable in nursing homes. The overall survival rate in people suffering OHCA in living facilities was 4.4% during the 31-year study period. There was an advantage of 1.0% in 30-days survival for private residences in the unadjusted analyses (*p* < 0.001), while the adjusted longitudinal model displayed a positive trend in annual survival odds in both private residences (5.6%) and in nursing homes (3.5%), with no difference between the groups (*p* = 0.207).

**Conclusions:**

In this registry study, 30-day survival in nursing homes and private residences was similar and improved in both settings. These findings suggest that the location of OHCA is not the primary determinant of survival. Resuscitation decisions should be guided by careful consideration of the patient’s medical condition, frailty, and personal preferences. Future initiatives might include strengthening emergency preparedness in nursing homes while supporting ethically justified and patient-centred shared decision-making.

**Trial registration:**

Not applicable.

**Supplementary Information:**

The online version contains supplementary material available at 10.1186/s13049-025-01496-y.

## Background

Out-of-hospital cardiac arrest (OHCA) is a global health problem with a high risk of mortality [1]. In Sweden, the total number of annual deaths is approximately 94 000 people [2] and according to the Swedish Register of Cardiopulmonary Resuscitation (SRCR), the number of attempted resuscitation attempts is approximately 8 500 annually. For 2023, 30-day survival rates in Sweden were 12.4% for OHCA and 36.5% for in-hospital cardiac arrest respectively. The number of people dying after OHCA and not receiving cardiopulmonary resuscitation (CPR) is unknown [3]. 

As in other Western countries, the Swedish population over 65 years has been steadily growing for the past 50 years [4, 5]. The number of people moving into nursing homes has increased since 2021 with most of them being over 80 years old. The median survival time after moving into a nursing home is 25 months, however with large variations between municipalities [6]. Some studies indicate that the incidence of OHCA is increasing, including people living in nursing homes [7–9]. 

Survival after OHCA depends on several factors where time to emergency call, CPR and defibrillation have a major impact. Most cardiac arrests occur out-of-hospital, and about two-thirds of these occur in private residences [10]. The proportion of bystander CPR has increased over the years [11] but some studies indicate that nursing home staff seem hesitant to start CPR before arrival of the ambulance service [12, 13]. 

Given this demographic shift, it is important to understand the occurrence of resuscitations attempts in nursing homes, the treatment provided, and the proportion of survivors. Although a recent study showed an overall trend of increased survival in Swedish OHCA for the last 30 years [11], it is still unclear what treatment has been given and what outcome has been achieved for the oldest general population and for people living in nursing homes. There is also a lack of knowledge on whether treatment and outcomes among those suffering OHCA in nursing homes differ from OHCA in private residences.

## Aim

The aim of the study was to describe OHCA occurrence, treatment, and 30-day survival in people aged 65 years or older in Swedish nursing homes, in comparison with private residences.

## Materials and methods

This retrospective registry study was based on data from the SRCR and was approved by the Swedish Ethical Review Authority (No 2023-04936-01).

### The Swedish register of cardiopulmonary resuscitation (SRCR)

The SRCR (https://shlr.registercentrum.se/) is a nationwide quality registry dedicated to the comprehensive documentation of cardiac arrest. All emergency hospitals and ambulance organizations across Sweden contribute data to the registry. Information is systematically recorded at the time of the cardiac arrest, capturing variables such as the location of the incident and the interventions provided [14]. When an OHCA occurs and resuscitation is initiated—regardless of the provider—and ambulance services are alerted, the event should be recorded in the SRCR by the ambulance staff. The first registration includes variables like e.g., witnessed status, initial rhythm, and treatment. The registration is supplemented, typically within a minimum of 30 days following the event, with additional data collection. This second phase includes variables, like e.g., comorbidities, post-arrest treatments, and survival at 30 days. Furthermore, survivors undergo a third follow-up between three to six months post-arrest including patient-reported outcome measures [14]. In this study we focused on people ≥ 65 years suffering OHCA from 1992 to 2022 in nursing homes and in private residences. Overall, more than 82 000 OHCAs were reported during 1992–2022. In Swedish healthcare, CPR must be started if there is no do-not-attempt cardiopulmonary resuscitation (DNACPR) order in place [15]. In SRCR, there are no data pertaining to DNACPR orders.

The OHCAs are documented by ambulance staff and then the registrations are evaluated by a local coordinator. The registration is based on the Utstein guidelines and describes characteristics, occurrence, treatment, and outcome. Variables include initial rhythm, categorized as either shockable (ventricular fibrillation, pulseless ventricular tachycardia) or non-shockable (pulseless electrical activity or asystole), time indications from collapse to emergency call, CPR, defibrillation, and ambulance arrival, treatment provided by the ambulance staff, e.g., mechanical chest compressions and medications, and admission to hospital. The main outcome is survival at 30 days [16]. In this study, aetiology was dichotomised to medical or non-medical causes, including all other causes like drowning or trauma, and medical drugs include both amiodarone and/or adrenaline. Ambulance witnessed cases were excluded when analysing bystander witnessed cases and time variables. Additionally, ambulance witnessed cases and missing data were excluded when presenting proportions of bystander CPR in results.

### Nursing homes in Sweden

Nursing homes, or residential facilities, are described as living facilities which provide nursing supervision and limited medical care to persons who do not require hospitalization [17]. It is a needs-based form of housing according to the Social Services Act (SFS, 2001:453) and can be considered both a residence, a care environment, and a workplace. In Swedish nursing homes, there should be access to staff around the clock [18]. Healthcare and medical interventions are carried out by both licenced professionals, such as nurses, and unlicensed care personnel, such as nursing assistants and care aides. When healthcare tasks are delegated, care personnel also fall under the Healthcare Act [19]. Primarily, care personnel provide care and support to older persons, which include assisting with personal hygiene, medication administration, meals and daily activities. The personnel play a crucial role in maintaining the well-being and quality of life of the elderly. Despite many lacking formal healthcare education, their practical experience and dedication are essential in addressing the complex needs of older persons with chronic diseases [6]. According to the Social Services Act [18], there are no legal obligations for staff working in nursing homes to initiate CPR, only recommendations [20]. According to recommendations by the Swedish Resuscitation Council, care personnel should undergo CPR training and repeat it annually [20]. This is in accordance with the European Resuscitation Council (ERC) guidelines on CPR training [21]. In most cases, medical interventions are performed by a primary care physician at a health or medical center. One of the primary care physician’s tasks is to determine whether CPR should be performed in the event of sudden cardiac arrest [6, 15, 22]. 

### Statistics

Variables were divided into two 10-year periods (1992–2001, 2002–2011) and one 11-year period (2012–2022). The division into periods was due to the extensive development of CPR training, AED availability and guidelines during the study period. The SRCR has been developed over time and some variables have therefore changed. Descriptive statistics were used to summarize data, including means, standard deviations (SD), interquartile ranges and percentages. To evaluate differences between groups, independent two-sample t-test, chi-square test, and two-proportion z-tests were applied depending on characteristics of the variable. All tests were two-tailed, and *p*-values of < 0.05 were considered statistically significant. Most statistical analyses were performed using IBM SPSS Statistics^®^ version 29 (Armonk, NY, IBM Corp). To estimate the annual change in occurrence, a generalized linear model with a quasi-Poisson distribution to account for overdispersion was used. The model was adjusted for age, sex, and aetiology. Similarly, to estimate the annual change in the odds of 30-day survival, a generalized linear model with a binomial distribution was used. The model was adjusted for age, sex, aetiology, and initial rhythm. The models were fitted using `glm` in R [23]. 

## Results

### Sample characteristics and occurrence of OHCA

**Fig. 1:**
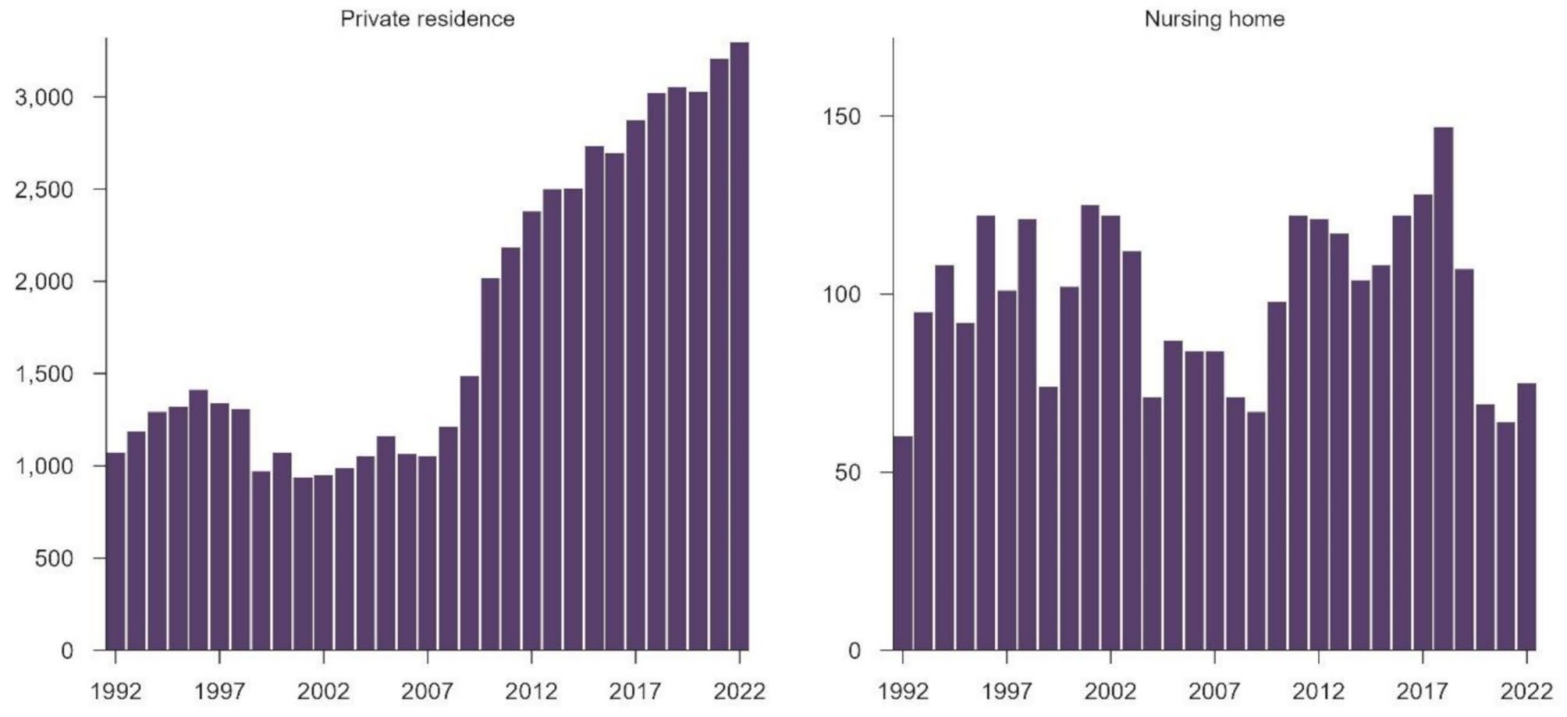
Occurrence of out-of-hospital cardiac arrest over a 31-year period

A total of 59 459 OHCAs occurred in nursing homes (*n* = 3 080) and in private residences (*n* = 56 379) during the study period, e.g., 1992–2022. The occurrence increased in private residences but remained stable in nursing homes since the start of the registry in 1990 (Fig. 1).

For the total sample, the mean age (SD) was 78.1 (± 7.7) and the majority (*n* = 36 628, 61.6%) were men. In nursing homes, the mean age (SD) was 81.6 (± 7.6) years compared to 77.9 (± 7.7) years in private residences. The distribution between the sexes was almost equal in nursing homes (48.6 vs. 51.4%), while the majority in private residences were men (63.0% vs. 37.0%, *p* < 0.001). The distribution of age and sex were stable over time. For the total sample, 37 203 (62.6%) had a witnessed OHCA, with a higher proportion in nursing homes compared to private residences (75.7% vs. 61.9%, *p* < 0.001). The proportion of witnessed cardiac arrests remained stable during the study period. The proportion of shockable initial rhythm has clearly decreased over time in both locations (*p* < 0.001), with a higher total proportion in private residences (19.9% vs. 12.3%, *p* < 0.001). Most patients, irrespective of location, had a medical cause underlying the arrest. More information about proportions and differences between groups within the different time periods are available in Table 1.

**Table 1:** Trends in characteristics of out-of-hospital cardiac arrest (*n* = 59 459) in relation to location

	1990–2001			2002–2011			2012–2022		
	Nursing homes	Private residences	*p*-value	Nursing homes	Private residences	*p*-value	Nursing homes	Private residences	*p*-value
**Occurrence** – n	1 000	11 921	< 0.001	918	13 166	< 0.001	1 162	31 292	< 0.001^*a*^
**Age**,** years** – mean (SD) min – max	81.57 (6.9) 65–98	76.39 (6.7) 65–99	< 0.001	81.49 (7.7) 65–102	77.73 (7.6) 65–107	< 0.001	81.92 (8.3) 65–104	78.59 (8.0) 65–106	< 0.001^*a*^
Missing – n (%)	16 (1.6)	0		13 (1.42)	0		26 (2.24)	0	
**Sex** – n (%)									
Male	443 (44.3)	7 767 (65.1)	< 0.001	445 (48.5)	8 429 (64.0)	< 0.001	579 (49.9)	18 965 (60.6)	< 0.001^*b*^
Female	518 (51.8)	3 683 (30.9)		452 (49.2)	4 600 (35.0)		582 (50.0)	12 315 (39.4)	
Missing	39 (3.9)	471 (4.0)		21 (2.3)	137 (1.0)		1 (0.1)	12 (0.0)	
**Witnessed arrest** – n (%)									
Yes	723 (72.3)	7 241 (60.7)	< 0.001	710 (77.3)	8 643 (65.6)	< 0.001	899 (77.4)	18 987 (60.7)	< 0.001^*b*^
No	186 (18.6)	3 711 (31.1)		163 (17.8)	3 828 (29.1)		245 (21.1)	11 195 (35.8)	
Missing	91 (9.1)	969 (8.1)		45 (4.9)	695 (5.3)		18 (1.5)	1 110 (3.5)	
**Presumed cause** – n (%)									
Medical	832 (83.2)	10 893 (91.4)	< 0.001	756 (82.4)	11 990 (91.1)	< 0.001	999 (86.0)	26 320 (84.1)	0.057^*b*^
Other	87 (8.7)	162 (1.3)		93 (10.1)	332 (2.5)		128 (11.0)	2 812 (9.0)	
Missing	81 (8.1)	866 (7.3)		69 (7.5)	844 (6.4)		35 (3.0)	2 160 (6.9)	
**First monitored rhythm** – n (%)									
			< 0.001			0.002			0.035^*c*^
Asystole	389 (38.9)	5 684 (47.7)		433 (47.2)	6 714 (51.0)		677 (58.3)	17 626 (56.3)	
PEA	112 (11.2)	1 086 (9.1)		189 (20.6)	1 860 (14.1)		277 (23.8)	5 507 (17.6)	
VT/VF	166 (16.6)	3 011 (25.3)		116 (12.6)	2 623 (20.0)		90 (7.7)	4 496 (14.4)	
Missing	333 (33.3)	2 140 (17.9)		180 (19.6)	1 969 (14.9)		118 (10.2)	3 663 (11.7)	

PEA = Pulseless Electrical Activity, VT = Ventricular Tachycardia, VF = Ventricular Fibrillation

^*a*^Independent two-sample t-test

^*b*^Two-proportion z-test

^*c*^Chi-square test

### Annual change of occurrence

In private residences, the number of OHCAs increased annually by 4.0% (95% CI: 3.8% – 4.3%; *p* < 0.001). In nursing homes, the increase was not statistically significant, corresponding to a near-stable trend (95% CI: -0.8% – 0.8%; *p* = 0.984). Compared to private residences, the increase in nursing homes was 4.0% slower (95% CI: -4.7% – -3%; *p* < 0.001).

### Treatment

Overall, the proportion of bystander CPR before ambulance arrival (57.5%) increased over time in both settings and was significantly higher in nursing homes compared to private residences (61.0% vs. 57.3%, *p* = 0.004). This means that the proportion of patients receiving bystander CPR in private residences increased from 28.1% to 49.9%, and subsequently to 83.7% over the course of the study period. The corresponding figures for nursing homes were 41.8%, 56.6%, and 89.2%, respectively. The median (IQR) time from event to emergency call was stable and significantly longer in nursing homes; 2 (4) vs. 2 (4) min, *p* < 0.001, however not reflected by median (IQR). The time to initiating CPR decreased consistently and was significantly shorter in nursing homes; 4 (10) vs. 7 (11) min, *p* < 0.001. The time to first defibrillation increased in both groups and was significantly shorter in nursing homes; 15 (12) vs. 17 (14) min, *p* < 0.001. The time to ambulance arrival was stable and significantly shorter in nursing homes; 11 (9) vs. 13 (11) min, *p* < 0.001. The use of mechanical chest compressions increased during the last 20 years, with a significantly lower proportion in nursing homes (17.7% vs. 27.2%, *p* = 0.007). The proportion of patients receiving ventilation increased in both groups with a significantly lower proportion in nursing homes (43.2% vs. 59.3%, *p* = 0.003). The proportion of patients receiving defibrillation by ambulance staff decreased longitudinally in both groups with a significantly lower proportion in nursing homes (22.9% vs. 31.9%, *p* < 0.001). The administration of medical drugs fluctuated longitudinally with a significantly lower proportion in nursing homes (72.0% vs. 77.0%, *p* < 0.001). In total, 14.3% of patients were admitted to hospital, with a higher proportion in nursing homes (15.5% vs. 14.3%, *p* = 0.384). More information about proportions and differences between groups within the different time periods are available in Table 2.

**Table 2:** Trends in treatment of out-of-hospital cardiac arrest (*n* = 59 459) in relation to location

	1992–2001			2002–2011			2012–2022		
	Nursing homes (*n* = 1 000)	Private residences (*n* = 11 921)	*p*-value	Nursing homes (*n* = 918)	Private residences (*n* = 13 166)	*p*-value	Nursing homes (*n* = 1 162)	Private residences (*n* = 31 292)	*p*-value
**Bystander CPR before ambulance arrival** – n (%)*									
Yes	231 (23.1)	1 745 (14.6)	< 0.001	321 (35.0)	3 486 (26.5)	< 0.001	413 (35.5)	7 367 (23.5)	0.020^*a*^
No	321 (32.1)	4 460 (37.4)		246 (26.8)	3 506 (26.6)		50 (4.3)	1 435 (4.6)	
Excluded (ambulance witnessed)	153 (15.3)	847 (7.1)		132 (14.4)	1 452 (11.0)		135 (11.6)	3 349 (10.7)	
Missing	295 (29.5)	4 869 (40.8)		219 (23.8)	4 722 (35.9)		564 (48.5)	19 141 (61.2)	
**Time from event in minutes** – median (IQR)*									
Time to emergency call	-	-		1 (3)	2 (4)	0.069	2 (4)	1 (4)	< 0.001^*b*^
Number of observations	-	-		143	2 668		654	13 382	
Time to start CPR	8 (10)	12 (10)	< 0.001	5 (10)	8 (12)	< 0.001	1 (5)	4 (9)	< 0.001^*b*^
Number of observations	377	4 944		416	5 806		669	13 342	
Time to first defibrillation	13 (11)	15 (12)	0.241	15 (10)	17 (14)	< 0.001	17 (12)	17 (15)	0.513^*b*^
Number of observations	142	2 839		128	2 713		118	4 690	
Time to ambulance arrival	10 (8)	12 (9)	< 0.001	10 (9)	12 (10)	< 0.001	12 (10)	13 (12)	< 0.001^*b*^
Number of observations	402	5 105		441	5 978		644	13 140	
**Ambulance treatment** – n (%)									
Mechanical CPR	-	-							
Yes				72 (7.8)	1 534 (11.7)	0.674	474 (40.8)	13 806 (44.1)	0.031^*a*^
No				204 (22.2)	4 095 (31.1)		624 (53.7)	15 901 (50.8)	
Missing				642 (70.0)	7 537 (57.2)		64 (5.5)	1 585 (5.1)	
Ventilation	-	-							
Yes				264 (28.8)	5 611 (42.6)	0.005	1 067 (91.8)	27 929 (89.3)	< 0.001^*a*^
No				13 (1.4)	121 (0.9)		76 (6.6)	2 988 (9.5)	
Missing				641 (69.8)	7 434 (56.5)		19 (1.6)	375 (1.2)	
Defibrillation									
Yes	291 (29.1)	5 173 (43.4)	< 0.001	227 (24.7)	4 638 (35.2)	< 0.001	186 (16.0)	8 142 (26.0)	< 0.001^*a*^
No	329 (32.9)	2 917 (24.5)		406 (44.2)	5 793 (44.0)		907 (78.1)	21 584 (69.0)	
Missing	380 (38.0)	3 831 (32.1)		285 (31.1)	2 735 (20.8)		69 (5.9)	1 566 (5.0)	
Medical drugs									
Yes	616 (61.6)	7 773 (65.2)	0.022	682 (74.3)	10 629 (80.7)	< 0.001	920 (79.2)	24 990 (79.9)	0.379^*a*^
No	384 (38.4)	4 148 (34.8)		235 (25.6)	2 514 (19.1)		225 (19.3)	5 719 (18.3)	
Missing	0	0		1 (0.1)	23 (0.2)		17 (1.5)	583 (1.8)	
**Hospitalized** – n (%)⁑									
Yes	146 (14.6)	1 455 (12.2)	< 0.001	163 (17.8)	2 135 (16.2)	0.638	169 (14.5)	4 446 (14.2)	0.617^*a*^
No	693 (69.3)	9 508 (79.8)		420 (45.7)	5 755 (43.7)		287 (24.7)	7 186 (23.0)	
Missing	161 (16.1)	958 (8.0)		335 (36.5)	5 276 (40.1)		706 (60.8)	19 660 (62.8)	

CPR = Cardiopulmonary Resuscitation

^*a*^Two-proportion z-test

^*b*^Independent two-sample t-test

*Ambulance witnessed cases excluded

⁑Patients transported by ambulance to hospital

### Survival at 30 days

**Fig. 2:**
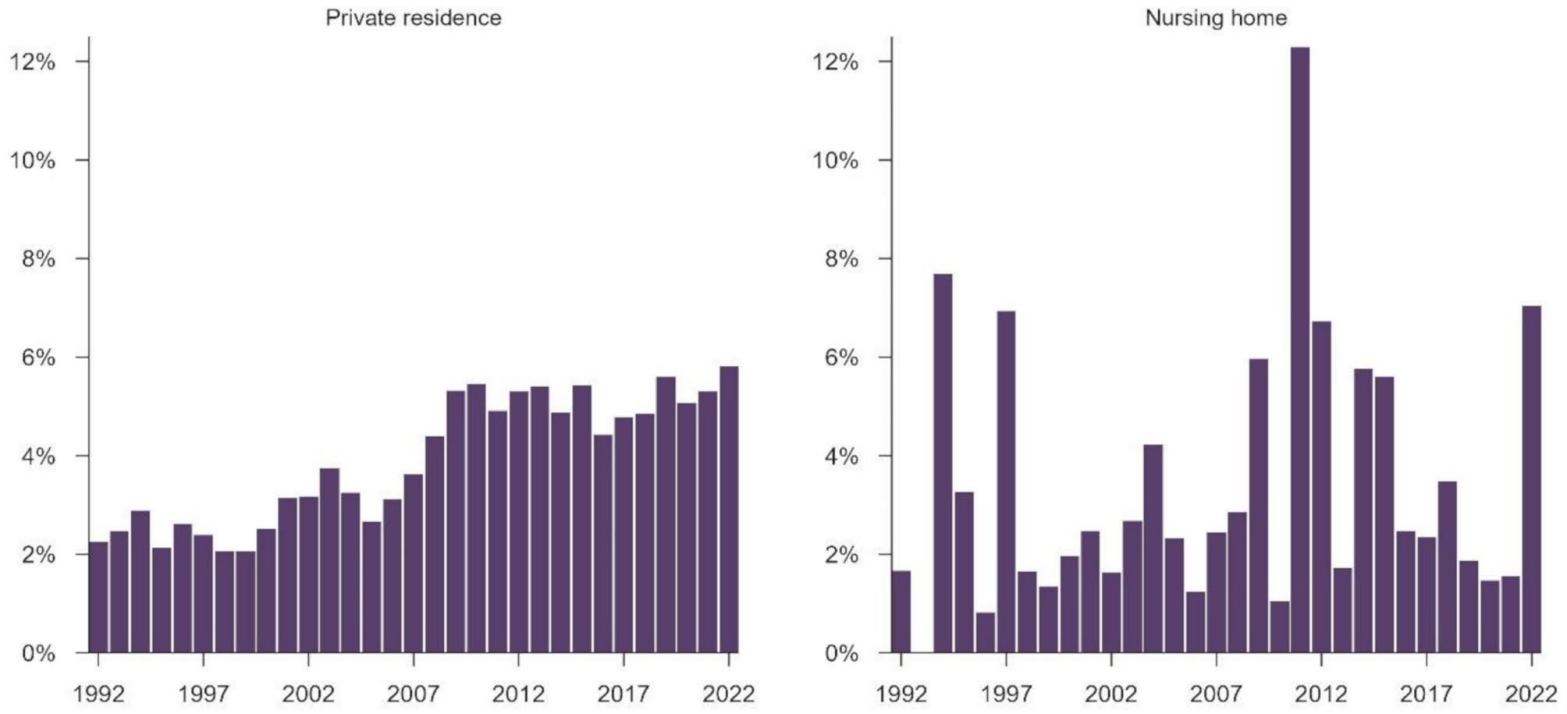
Out-of-hospital cardiac arrest survival at 30 days over a 31-year period

The overall survival rate at 30 days was 4.3% during the study period, with 3.4% vs. 4.4% in nursing homes and private residences respectively (*p* = 0.016). Survival rates improved over time in private residences. In nursing homes, the survival rate improved from the first to second time period, while deteriorated from the second to the third (Fig. 2; Table 3).

**Table 3:** Survival at 30 days

	1992–2001			2002–2011			2012–2022		
	Nursing homes (*n* = 1 000)	Private residences (*n* = 11 921)	*p*-value	Nursing homes (*n* = 918)	Private residences (*n* = 13 166)	*p*-value	Nursing homes (*n* = 1 162)	Private residences (*n* = 31 292)	*p*-value
Survival at 30 days, n (%)	28 (2.8)	290 (2.4)	0.459	35 (3.8)	552 (4.2)	0.610	42 (3.6)	1 612 (5.2)	0.021
Missing, n (%)	8 (0.8)	50 (0.4)		9 (1.0)	35 (0.3)		12 (1.0)	165 (0.5)	

All *p*-values are based on two-proportion z-test

### Annual change in survival

In private residences, the annual increase in survival odds was 5.6% (95% CI: 4.9%–6.2%; *p* < 0.001). In nursing homes, the corresponding increase was 3.5% (95% CI: 0.4%–6.6%; *p* = 0.025). Compared to private residences, the annual increase in nursing homes was 2.0% slower, although not statistically significant (95% CI: -5%–1.2%; *p* = 0.207).

## Discussion

The overall survival rate in people aged ≥ 65 suffering OHCA in living facilities was low during the 31-year study period. Even though there was a statistically significant advantage of 1.0% in 30-days survival for private residences in the unadjusted analyses, the longitudinal adjusted model displayed no difference in survival. As expected, we found a positive trend in survival in both private residences and nursing homes. In addition, the occurrence of OHCA increased in private residences but remained stable in nursing homes. Finally, there were some significant differences between the groups in time to critical treatments.

The overall survival rate in the present study was low compared to an international review [1] and a study by Jerkeman et al., also based on data from the SRCR, which reported survival rates between 5% and 11% [11]. This discrepancy can partly be explained by differences in sample characteristics. The population in our study was people ≥ 65 years suffering OHCA in living facilities, while Jerkeman’s study included all OHCAs regardless of age and location [11]. The lower survival rates observed in our study may also be attributable to the higher prevalence of comorbidities resulting in poorer prognosis. A recent Norwegian study on OHCAs among individuals ≥ 60 years reported lower survival rates in healthcare institutions (4.8%) compared with private homes (8.5%). However, the study encompassed a wide range of care settings rather than focusing exclusively on nursing homes. These differences in population and care context limit direct comparability with our findings, which specifically address nursing home residents. Variations in care level, staffing, and patient characteristics across settings may influence both treatment, decisions and outcomes [24]. Over the past decades, significant advancements have been achieved in OHCA response, including publicly accessible AEDs, CPR awareness in the community, and volunteer responder systems. However, previous studies have reported limited use of AEDs prior to ambulance arrival for residential OHCAs, especially in nursing homes. The main reason for this is probably the lack of available AEDs [25, 26]. In the present study, we had no valid data on AED availability or use. This should be further explored.

In the present study, the overall 30-day survival rate in nursing homes was 3.4% and lower compared to private residences while previous studies from other countries have reported survival rates between 0.3% and 2.6% in nursing homes [26–31]. Compared to these studies our findings indicate a higher chance of survival in Swedish nursing homes. However, the variation between our study and earlier studies is likely influenced by multiple factors, including demographic characteristics, healthcare accessibility, emergency response, and cultural differences regarding end-of-life decision making. The definition of a nursing home may also differ depending on the context [17]. Although we found a 1.0% higher chance of survival in private residences the clinical importance may be discussed. A significant unadjusted *p*-value and related clinical implications should be interpreted with great caution in a large sample study, with a high risk of making a type I error. Importantly, in the adjusted longitudinal model we found no differences between the groups.

There was a difference in occurrence of OHCA between private residences and nursing homes. In private residences, the increasing annual occurrence might be associated with e.g., enhanced emergency response systems and improved CPR awareness in the community. On the other hand, the stable trend in nursing homes may reflect differences in patient frailty, patient preferences, or attitudes of professionals. Given the limited occurrence of OHCAs in nursing homes it may be important to further investigate the use of advance care planning and do-not-attempt cardiopulmonary resuscitation (DNACPR) orders in this setting.

In a study about healthcare professionals’ perceptions of futile CPR, two thirds of the respondents believed that resuscitation attempts were inappropriate for people living in nursing homes [32]. When it comes to discussing CPR with elderly people, frailty needs to be considered in terms of survival prognosis. This is supported by a meta-analysis concluding that increased frailty resulted in a markedly increased mortality in those receiving CPR [33]. It is likely that people suffering OHCA in nursing homes are more frail compared to those in private residences, which might affect both occurrence and outcomes since frail people are more likely to have a DNACPR decision and a lower chance of survival when CPR is initiated. To ensure that resuscitation efforts align with individual values and clinical realities, there might be a need to strengthen the implementation of structured advance care planning, including timely and transparent DNACPR decision-making [22]. Future research should therefore explore not only survival, but also patient-centered outcomes such as quality of life, functional recovery, and the extent to which care aligns with individual preferences. Such an approach would provide a more balanced and ethically grounded understanding of CPR in the present population.

Throughout the study period, there was a remarkable increase in the proportion of patients receiving bystander CPR in both settings. Although patients suffering OHCA in nursing homes were more likely to be witnessed, receiving bystander CPR, and having shorter time to ambulance arrival, the chance of survival was similar as in private residences. This suggests that other factors influence the likelihood of survival. Those suffering OHCA in private residences more often had a shockable initial rhythm and received more defibrillations. Therefore, since shockable initial rhythm is a strong positive predictor for survival [34, 35], the difference is likely to influence our findings. Moreover, previous research suggests that CPR training may be insufficient in nursing homes [12, 13, 32], which could be another influential factor for survival. Healthcare staff lacking regular CPR training do not retain their skills, which might negatively affect patient outcome [36, 37]. In a recent Danish study, OHCAs in nursing homes with optimal conditions, i.e., being bystander witnessed, and treated with CPR and defibrillation, were predicted to have a 30-day survival of 7.7% [27]. Therefore, according to recently published Swedish guidelines, it is crucial to standardize CPR training and ensure that it is tailored to the needs of nursing home staff to improve patient outcomes [20]. 

Interestingly, and contrary to previous Swedish research [11], our data indicate that the time from OHCA to ambulance arrival remained stable throughout the study period. This unexpected finding warrants further investigation.

### Limitations

The study encompasses nearly 60 000 cases over a span of three decades, providing a comprehensive longitudinal overview of ambulance-treated OHCAs in the older population in Sweden. However, this study has some limitations, primarily due to a high proportion of missing data for some variables, e.g., hospitalization and time variables. Therefore, results based on these variables should be interpreted with caution. The main reason for missing data was that these variables were not consistently included in the registry throughout the entire study period. However, it is important to note that the main outcome—30-day survival—had a low proportion of missing data (0.5%), which strengthens the reliability of the primary findings (supplementary file 1).

Some cases in this study occurred during the COVID-19 pandemic (2020–2022), a period that may have affected occurrence, treatment, and outcomes of OHCA due to changes in healthcare delivery, emergency response, and resuscitation practices. This aspect should be considered when interpreting the findings.

Additionally, the registry does not capture data on patients who died without receiving CPR, and therefore they are not included in this study. Furthermore, information on patients’ co-morbidities, frailty, and prior functional status was unavailable, as these variables were not recorded. This limits the ability to assess the appropriateness of CPR and the potential for meaningful recovery. This should be considered when interpreting the findings. These factors are important for a more nuanced interpretation of the findings and should be further investigated. Further, calculation of incidence would have contributed to more robust results than occurrence. However, we did not have access to such data. Also, data on ROSC and treatment duration were not available. Such information could have provided valuable insights into the decision-making process. Finally, the registry aims to include all patients who receive CPR, regardless of who administers the treatment. However, we recognise that there may be gaps in compliance, which could result in cases being missed—particularly when CPR is not provided directly by ambulance staff.

## Conclusion

In this registry study, 30-day survival in nursing homes and private residences was similar and the longitudinal survival improved in both settings over the 31-year period. These findings suggest that the location of OHCA is not the primary determinant of survival. Resuscitation decisions should be guided by careful consideration of the patient’s medical condition, frailty, and personal preferences. However, whether such considerations are taken needs further investigation. Future initiatives might include strengthening emergency preparedness in nursing homes while supporting ethically justified and patient-centred shared decision-making. Additionally, the underlying reasons for the relatively low occurrence of OHCA in nursing homes warrant further investigation.

## Supplementary Information

Below is the link to the electronic supplementary material.

Supplementary Material 1

## Data Availability

The datasets used and analysed in this study are derived from healthcare records and contain sensitive patient information. Due to ethical considerations and legal restrictions under the General Data Protection Regulation (GDPR), these data cannot be made publicly available. Access to anonymized or aggregated data may be considered upon reasonable request to the corresponding author, subject to approval by the relevant ethics committee and data custodian.

## Abbreviations

AED: Automated External Defibrillator
CPR: Cardiopulmonary resuscitation
DNACPR: Do–not–attempt cardiopulmonary resuscitation
OHCA: Out–of–hospital cardiac arrest
SRCR: Swedish Registry of Cardiopulmonary Resuscitation
