# Supplementary material for: Out-of-hospital cardiac arrests in Swedish nursing homes: occurrence, treatment, and survival compared to private residences
Source: Scand J Trauma Resusc Emerg Med. 2025 Oct 21;33:170. doi: 10.1186/s13049-025-01496-y (PMC12538722; doi:10.1186/s13049-025-01496-y)
Supplement: Supplementary file 1 — Supplementary Material 1 [file 13049_2025_1496_MOESM1_ESM.docx]

| **Supplementary file 1** Number and proportion of missing data | | |
| --- | --- | --- |
| Variabels |  | Total number of missing values (% of 59 459) |
| Time from event to defibrillation^†^ |  | 43 691 (73.5)^a^ |
| Time from event to emergency call |  | 29 133 (49.0)^b^ |
| Hospitalized |  | 27 096 (45.6)^a^ |
| Mechanical compressions |  | 22 749 (38.3)^b^ |
| Ventilation by ambulance |  | 21 390 (36.0)^b^ |
| Bystander CPR |  | 19 018 (32.0)^a^ |
| Time from event to start of CPR |  | 15 125 (25.4)^a^ |
| Time from event to ambulance arrival |  | 14 927 (25.1)^b^ |
| Initial rhythm |  | 8 373 (14.1)^a^ |
| Probable cause |  | 4 055 (6.8)^c^ |
| Witnessed |  | 2 928 (4.9)^a^ |
| Sex |  | 681 (1.1)^a^ |
| Medical drugs |  | 624 (1.0)^a^ |
| Survival at 30-days |  | 279 (0.5)^a^ |
| Age |  | 55 (0.1)^a^ |
| Variables available in registry from: ^a^1990-, ^b^2007-, ^c^1990-2020.  ^†^ Missing values for time from event to defibrillation includes patients not receiving defibrillation | | |
